# Frequent Missense and Insertion/Deletion Polymorphisms in the Ovine *Shadoo* Gene Parallel Species-Specific Variation in PrP

**DOI:** 10.1371/journal.pone.0006538

**Published:** 2009-08-06

**Authors:** Nathalie Daude, Serene Wohlgemuth, Ekaterina Rogaeva, A. Hossein Farid, Mike Heaton, David Westaway

**Affiliations:** 1 Centre for Prions and Protein Folding Diseases, University of Alberta, Edmonton, Alberta, Canada; 2 Centre for Research in Neurodegenerative Diseases, University of Toronto, Toronto, Ontario, Canada; 3 Department of Animal Science, Nova Scotia Agricultural College, Truro, Nova Scotia, Canada; 4 United States Department of Agriculture, U. S. Meat Animal Research Center, Clay Center, Nebraska, United States of America; Université de Toulouse, France

## Abstract

**Background:**

The cellular prion protein PrP^C^ is encoded by the *Prnp* gene. This protein is expressed in the central nervous system (CNS) and serves as a precursor to the misfolded PrP^Sc^ isoform in prion diseases. The prototype prion disease is scrapie in sheep, and whereas *Prnp* exhibits common missense polymorphisms for V136A, R154H and Q171R in ovine populations, genetic variation in mouse *Prnp* is limited. Recently the CNS glycoprotein Shadoo (Sho) has been shown to resemble PrP^C^ both in a central hydrophobic domain and in activity in a toxicity assay performed in cerebellar neurons. Sho protein levels are reduced in prion infections in rodents. Prompted by these properties of the Sho protein we investigated the extent of natural variation in *SPRN*.

**Principal Findings:**

Paralleling the case for ovine versus human and murine *PRNP*, we failed to detect significant coding polymorphisms that alter the mature Sho protein in a sample of neurologically normal humans, or in diverse strains of mice. However, ovine *SPRN* exhibited 4 missense mutations and expansion/contraction in a series of 5 tandem Ala/Gly-containing repeats R1-R5 encoding Sho's hydrophobic domain. A Val71Ala polymorphism and polymorphic expansion of wt 67(Ala)_3_Gly70 to 67(Ala)_5_Gly72 reached frequencies of 20%, with other alleles including Δ67–70 and a 67(Ala)_6_Gly73 expansion. Sheep V71, A71, Δ67–70 and 67(Ala)_6_Gly73 *SPRN* alleles encoded proteins with similar stability and posttranslational processing in transfected neuroblastoma cells.

**Significance:**

Frequent coding polymorphisms are a hallmark of the sheep *PRNP* gene and our data indicate a similar situation applies to ovine *SPRN*. Whether a common selection pressure balances diversity at both loci remains to be established.

## Introduction

Prion diseases (also known as Transmissible Spongiform Encephalopathies, “TSE”s) are a group of fatal neurodegenerative diseases that include Creutzfeldt-Jakob disease (CJD), scrapie and bovine spongiform encephalopathy (BSE) in human, sheep and cattle, respectively [1]. The pathological hallmarks of these diseases include spongiform change of the neuropil and accumulation of misfolded forms (“PrP^Sc^”, PrP-scrapie or “PrP^D^” to denote PrP-disease) of the cellular prion protein (PrP^C^), with some but not all aggregates being visible by light microscopy in the form of amyloid deposits detected with intercalating dyes or PrP-directed antibodies [2]. Non-conserved missense mutations and insertions that affect the *PRNP* gene, which encodes the PrP protein, are associated with inherited human prion diseases. Moreover, polymorphisms at codon 129 (the major polymorphic site of the *PRNP* gene, present in many ethnic groups) influence susceptibility to the disease as well as the age of onset, clinical phenotype and the duration of the illness [3]. Genetic control of prion disease has also been demonstrated in animals. For example, missense polymorphisms in sheep at codons 136, 154 and 171 are strongly correlated with disease susceptibility and progression in animals affected by natural scrapie or experimental scrapie disease caused by administration of infectious inocula [4]–[7]. Sheep *PRNP* alleles conferring heightened susceptibility to natural scrapie disease may improve postnatal survival [8], offering a parallel to the literature defining neuroprotective properties for PrP^C^, and perhaps explaining the paradox of their retention in commercial stocks.

In addition to the obvious importance of the *PRNP* gene in modulating prion diseases, there are clinical and experimental findings that demonstrate that other factors play a role in disease pathogenesis. Recently, a gene denoted *SPRN*, coding for the protein Shadoo (Sho) has been identified as a new CNS-expressed member of the prion protein superfamily, a family now currently comprising PrP, Doppel and Sho [9]. Sho, like PrP, is GPI-anchored, N-glycosylated and endoproteolytically processed to a “C1” fragment. In a cerebellar granule cell assay, Sho has neuroprotective properties and is a plausible candidate for the hypothetical PrP^C^-like protein π (‘pi’) deduced from the phenotypic properties of PrP null (*Prnp* ^0/0^) and ΔPrP transgenic mice [10], [11]. Moreover, Sho protein levels are profoundly reduced in the brains of prion-infected rodents [11]. Thus, a detailed analysis of *SPRN* in different populations may be informative in determining its importance in physiology, and ultimately in the case of prion disease. Here we assess polymorphisms of *SPRN* in human and sheep populations, and inbred mouse strains. Our data define species-dependent levels of polymorphic variation and reveal potential DNA instability in the centre of the ovine *SPRN* coding sequence.

## Materials and Methods

### DNA samples

Mouse (Mus Musculus) DNA samples represent a panel of strains commonly used in a research laboratory. The different strains are: Swiss lineage (SWR/J, SJL/J, FVB/NCR), Castle's lineage (LP/J, 129S6, VM/DK, DBA/IJ, C3H/He SnJ, CBA/NJ, NZB/BinJ, NZW/lacJ, MA/MyJ, P/J), C57 lineage (C57l/J, C58/J), wild lineages (Molf/Ei, Cast, IS/Cam). Other lineages include RIIIS/J, RIII/DM, LG/J, and JE/Le, while Mus Spretus represents a related species. Sheep DNA samples were selected from DNA banks from Canadian and American sheep breeds. In addition to 10 two-generation unrelated sheep from North Country and Border Cheviots from different farms in British Columbia and Nova Scotia, the USMARC Sheep Diversity Panel version 2.4 (MSDP2.4) was used. The latter consists of samples from 96 rams representing nine popular US breeds of sheep and one Navajo-Churro ram. The latter ram was included because it has a rare A136, R154, K171 *PRNP* genotype. The nine major breeds are divided into four classifications: 1) general purpose breeds including Dorset, Rambouillet, and Texel; 2) terminal-sire breeds including Suffolk and Composite (1/4 Suffolk, 1/4 Hampshire, and 1/2 Columbia); 3) prolific breeds including Finnsheep and Romanov; and 4) hair-shedding breeds including Dorper and Katahdin. The sires within breeds were selected for minimal relationships within and between pedigrees. The samples were collected between 1997 and 2003 from rams that were two to five years old. Unrelated human DNA samples (n = 93) were collected in Toronto, have a predominantly Northern European origin, and were from individuals neurologically normal at the time of blood collection. Written informed consent for research purposes, approved by the University of Toronto research ethics board, was obtained from all individuals involved in the study.

### Cloning of a 4 kb sheep SPRN fragment

**Figure 1:**
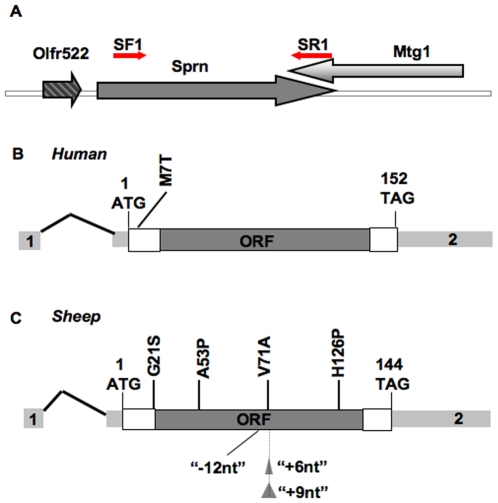
Overview of *SPRN* genes. (A) Consensus chromosomal structure of mammalian *SPRN* deduced from mouse and human prototypes: *SPRN* is flanked by the Olfr322 and Mtg1 genes. Oligonucleotide primers SF1 and SR1 used to amplify sheep *SPRN* sequences were deduced from this consensus structure. Panels B and C (not to same scale as Panel A) represent mRNAs encoding Human (B) and (C) sheep *SPRN*. Light grey boxes 1 and 2 represent the exons. The entire ORF (dark grey) of *SPRN* is located on exon 2. The common polymorphisms discussed in the main text are represented, as well as the indel variations (not to scale) in ovine *SPRN*.

The forward primer (SF1, **Table S1**) was designed upstream of the bovine open reading frame (ORF) of *SPRN* and the reverse primer (SR1, **Table S1**) was designed downstream of the sheep MGT1 sequence using the software Primerselect™ (**Figure 1**). Primer pairs were checked for hairpin and duplex formation and synthesized by Integrated DNA Technologies (Coralville, USA). PCR was performed in 25 µl volume containing 1 µM dNTPs, 0.2 µM of each primer, 5% DMSO, 1X reaction buffer and 1U of Accuprime™ Taq DNA polymerase (Invitrogen). Thermal cycling was performed on a Mastercycler® ep (Eppendorf) with a silver heating block, with the protocol: (1) 96°C for 5 min, (2) 25 cycles of 96°C for 30 s, 58°C for 30 s, and 72°C for 5 min, (3) 72°C for 10 min. PCR products were electrophoresed on agarose gels and visualized by SYBR® green staining and ultraviolet transillumination. For analysis of insertion/deletion (in/del) variations PCR products were analyzed on 15% (29∶1 acrylamide; bis-acrylamide) polyacrylamide gels run in 50 mM Tris-Borate EDTA buffer.

### Amplification of SPRN open reading frames

Primers used to amplify the mouse (MF1 and MR1), sheep (SF2 and SR2) and human (HF1 and HR1) (**Table S1**) ORFs were designed as previously described. The entire sheep ORF was amplified using the protocol: (1) 96°C for 5 min, (2) 35 cycles of 96°C for 30 s, 62°C for 30 s, and 72°C for 1 min, (3) 72°C for 10 min. For amplification of the mouse and human ORF chain elongation were extended to 90 sec.

### Sequencing and Bioinformatics

*SPRN* polymorphisms were detected by automated DNA sequencing and analysed by capillary electrophoresis on an ABI Prism 3730 DNA Analyzer (Applied Biosystems, Foster City, CA, USA). The sequencing primers, MF1, MR1, HF2, HR2, SF2 and SR2 (**Table S1**), were used for the mouse, human and sheep samples respectively. The reaction was set up according to the manufacturer's protocol using ‘Big Dye’ terminator chemistry. A final concentration of 5% DMSO and 1M betaine was added to the reaction mix to reduce potential secondary structure (because the high GC content of *SPRN*: see main text). Data were analysed using SeqMan™ software. When necessary (see main text), some PCR amplified samples were sub-cloned into pCR2.1 TOPO vector (Invitrogen) to confirm the chromosomal phase of nucleotide polymorphisms. The sheep genome database was interrogated with ISGC BLAST using the most current assembly.

### Expression of Sho plasmids in N2a neuroblastoma cells

The sheep Sho open reading frames (ORFs) of the alleles A71, V71, “+9”, and “−12” were amplified by PCR with primers SF3 and SR3 (Table S1) and cloned into the *Hin*dIII and *Xba*I sites of pBUD.CE4 also expressing GFP from a second promoter (Invitrogen). Sho mutants were generated using previous methods [12] with constructs verified by DNA sequencing. N2a cells were cultured, transfected and lysed as previously described [12]. 24 hours post-transfection, cells were treated with 35 µg/ml cycloheximide for 2 to 8 hours. 50 µg of total protein were treated by PNGaseI (New England Biolabs) for 4 hours. Protein was separated by SDS–PAGE using 12% polyacrylamide gels and transferred to nitrocellulose (5% non-fat skim milk block). Blots were incubated overnight with primary antibodies (06rSH-3a Sho polyclonals [11] or anti-GFP (Clontech) monoclonal, at 1∶1000 and 1∶500 respectively), incubated with HRP-conjugated secondary antibody and then developed using ‘Western Lightning’ ECL (Perkin-Elmer).

## Results

### Mouse *SPRN* polymorphisms

Genomic DNA from 22 diverse mice strains (see Materials and Methods) was examined for polymorphisms in the *SPRN* open reading frame. 22 samples did not present any polymorphisms and were thus similar to the published sequences (GI:56118240), and in agreement with NCBI SNP database entries available at time of writing. One further sample from wild *sprettus* mouse, comprising a different species from *Mus musculus* [13], exhibited polymorphisms (A/G at position 33, G/A at position 324 and T/C at position 408). Although these polymorphisms do not result in changes in the corresponding amino acids (Leu, Gly and Gly at codons 11, 108 and 136, respectively), a fourth polymorphism at position 400 (T/C) does results, in an amino acid change, L134F in the GPI attachment signal peptide.

### Human *SPRN* polymorphisms

Genomic DNA from 93 healthy human subjects was examined for polymorphisms in the uninterrupted *SPRN* open reading frame (**Table 1**). By virtue of i) selective primers, ii) the size of the PCR product used for sequencing reactions, and iii) the nature of the retrieved sequences we could absolutely assign these sequences to the authentic *SPRN* ORF and not the degenerated ORF of the *SPRN* pseudogene located 160 kb distally [14]. At position −11 relative to the start codon of the human *SPRN* gene, 35 subjects (37.6%) were genotyped as A/A, 14 subjects (15.1%) as G/G, and 44 subjects (47.3%) as A/G. The allele frequency is 62% for A. Two polymorphisms were identified in the ORF. The first causes the amino acid change T7M in the N-terminal signal peptide and is common, while a less frequent second polymorphism at codon 61 is silent. These two polymorphisms had previously been identified in the human single nucleotide polymorphism (SNP) database as rs 2492666 and rs 4077586, respectively. At nucleotide position 20 of the ORF (codon 7, signal peptide) the C allele reaches a frequency is 35% while a silent polymorphism at nucleotide position 183 (codon 61, T nucleotide) reaches a frequency of 29% **(** **Table 1** **)**. In sum, these data indicate a paucity of human missense polymorphisms affecting the mature Sho protein (i.e., the region that remains subsequent to endoproteolytic processing to remove the N- and C-terminal signal peptides).

**Table 1:** Human *SPRN* polymorphisms.

Position	−11		T7M		G61G	
**Genotype (%)**	A/A	37.6	T/T	45.2	C/C	49.4
	G/G	15.1	C/T	39.8	C/T	43
	A/G	47.3	C/C	15.1	T/T	7.5
**Predominant allele**	A = 61.7%		T = 65.1%		C = 71.0%	

### Cloning the ovine *SPRN* gene

While human *SPRN* sequences were available from genomic databases, an anchored PCR cloning strategy was employed to retrieve a prototype of the sheep *SPRN* gene. This strategy was based upon (i) the assumption that the ORF and the extended flanking regions would be highly conserved between cattle and sheep and (ii) a published sequence for the sheep *MTG1* gene, a gene that lies immediately adjacent (3′) to *SPRN* in other mammals. Thus, the bovine *SPRN* genome sequence was used as a template to design the forward primers to amplify the homologous region in sheep, with reverse primers deriving from the sheep *MTG1* sequence (**Figure 1A**). The resulting 3352 bp fragment of sheep *SPRN* was amplified, cloned and sequenced. Prior to the publication of this work a sequence similar to the wt sheep *SPRN* sequence was published by Lampo *et al* [Genbank (gi|145688401)[15]]. However this sequence deviates from the common prototype in sheep and other mammals in that it contains an additional 6 nucleotides and does not represent the most prevalent allelic type in our sample set. It also does not correspond to the number of unit repeats found in the wt SPRN genes of other mammalian species (discussed below).

### Common SNPs and trinucleotide indels in the ovine *SPRN* gene

#### Data overview

**Figure 2:**
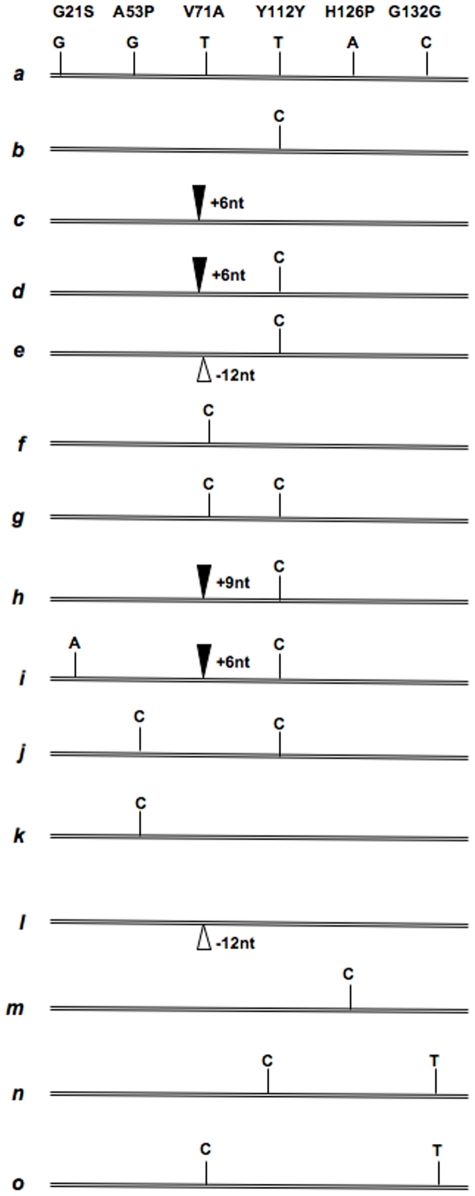
Sheep *SPRN* coding region haplotypes. Representation of the major coding region haplotypes observed in common sheep breeds. *a* and *b*, putative wild type ancestral haplotype and silent Y112Y polymorphism with a C residue in this codon, respectively; *c, d*, 6 bases insertion variants (solid fill triangle) of *a* and *b*, respectively: *e*, “−12” nucleotide deletion haplotype (open triangle); *f* haplotype encoding alanine at codon 71 and *g* same with silent Y112Y polymorphism with a C residue in this codon. Haplotypes *h* to *o* comprises less common variants represented by 3 or fewer animals.

A total of 107 DNA samples from healthy sheep were analyzed for *SPRN* polymorphisms. The samples were derived from two complementary sources. First, a panel of DNAs from a purebred stock of Cheviot, this breed being selected because it was the source for a wide variety of scrapie isolates deriving from the so-called “SSBP/1” inoculum [16]. The second source was from a pre-assembled “diversity panel” designed to represent the breadth of genotypic variation within the US sheep industry, comprising ten purebred sheep breeds and also a “composite” stock derived from interbreeding of Suffolk (1/4), Hampshire (1/2) and Columbia (1/4) breeds. Deduced allele types and genotype frequencies for the sheep *SPRN* gene are summarized in **Tables 2** **–** [Table pone-0006538-t003] **4**, and in **Figures 1** and **2**. In overview we found that the sheep *SPRN* coding region exhibits a variety of coding polymorphisms (see **Tables 2**). These polymorphisms fall into two broad categories. The first category corresponds to missense changes throughout the coding region, the most prevalent being V71A, and the second category to a series of in-frame trinucleotide indel variations in the vicinity of codon 70. Along with silent nucleotide replacements, the common nucleotide variations polymorphisms could be arranged into at least 15 coding region haplotypes as deduced by direct sequencing of PCR amplified DNA and also by cloning and sequencing of individual PCR products from certain heterozygous animals (**Figures 1** **,** **2**).

**Table 2:** Polymorphisms by sheep breeds.

# Animals	Breed	Codon	G21S		A53P		V71A		Y112Y		H126P		G132G		Indel
20	**Cheviot**	G/G	100	G/G	85.00	T/T	65	T/T	55	A/A	100	C/C	95	s/s	80
				G/C	15.00	C/C	5	C/C	5			C/T	5	“+6”/s	20
						C/T	30	C/T	40						
16	**Composite**	G/G	100	G/G	100	T/T	56.25	T/T	31.25	A/A	100	C/C	100	s/s	56.25
						C/T	43.75	C/C	18.75					“+6”/s	37.5
								C/T	50					“−12”/*	6.25
9	**Dorper**	G/G	100	G/G	100	T/T	100	T/T	77.78	A/A	100	C/C	100	s/s	77.78
								C/T	22.22					“+6”/s	22.22
9	**Dorset**	G/G	100	G/G	100	T/T	88.89	T/T	22.22	A/A	100	C/C	100	s/s	11.11
						C/T	11.11	C/C	44.44					“+6/+6”	33.33
								C/T	33.33					+6/s	55.56
9	**Finn**	G/G	100	G/G	100	T/T	100	T/T	22.22	A/A	100	C/C	100	s/s	44.44
								C/C	33.33					“+6”/s	22.22
								C/T	44.44					“−12”/*	33.33
7	**Katahdin**	G/G	85.71	G/G	100	T/T	85.71	T/T	14.29	A/A	85.71	C/C	71.43	s/s	42.86
		A/G	14.29			C/T	14.29	C/C	42.86	A/C	14.29	C/T	28.57	“+6/+6”	28.57
								C/T	42.86					“+6”/s	28.57
10	**Rambouillet**	G/G	100	G/G	100	T/T	90	T/T	70	A/A	90	C/C	100	s/s	60
						C/C	10	C/C	10	C/C	10			“+6”/s	40
								C/T	20						
10	**Romanov**	G/G	100	G/G	100	T/T	100	T/T	90	A/A	100	C/C	100	s/s	70
								C/C	10					“+6/+6”	10
														“+6”/s	20
7	**Suffolk**	G/G	100	G/G	100	T/T	85.71	T/T	57.14	A/A	85.71	C/C	100	s/s	85.71
						C/T	14.29	C/T	42.86	C/C	14.29			“+6”/s	14.29
9	**Texel**	G/G	100	G/G	100	T/T	77.78	T/T	44.44	A/A	100	C/C	100	s/s	77.78
						C/C	11.11	C/C	33.33					“+6”/s	22.22
						C/T	11.11	C/T	22.22						

* : either s (short) or “+6”.

**Table 3 pone-0006538-t003:** Overall frequencies of single nucleotide polymorphisms of sheep *SPRN*.

Codon	G21S		A53P		V71A		Y112Y		H126P		G132G	
**Genotype (%)**	G/G	99.1	G/G	97.2	T/T	81.3	T/T	48.6	A/A	97.2	C/C	97.2
	A/G	0.9	G/C	2.8	C/T	15.9	C/T	33.6	C/C	1.9	C/T	2.80
					C/C	2.8	C/C	17.8	A/C	0.9		
**Predominant allele**	G = 99.5%		G = 98.6%		T = 89.3%		T = 65.4%		A = 97.7%		C = 98.6%	

**Table 4:** Overall frequencies of sheep *SPRN* Indel polymorphisms.

	Wt	“−12”nt	“+6”nt	“+9”nt
**Homozygous (%)**	61.7		5.6	
**Heterozygous for indel (%)**		3.7	28.0	0.9
**Allele (%)**	77.6	1.9	20.0	0.5

### Missense polymorphisms of ovine *SPRN*

Overall, we found two frequent single base-pair polymorphisms, in codons 71 and 112 (V71A and Y112Y). There were 3 rare missense polymorphisms present in 5 or fewer alleles of the total sample of 214 alleles (**Table 3**: G21S, A53P, H126P), and also a fourth silent polymorphism G132G. Note that the codon numbering scheme used here and below is based upon our establishment of the most frequent “*a*” allele of *SPRN* as a likely prototype of the ancestral ovine sequence (Genbank #EU380591).

#### Prevalent SNPs

**Figure 3:**
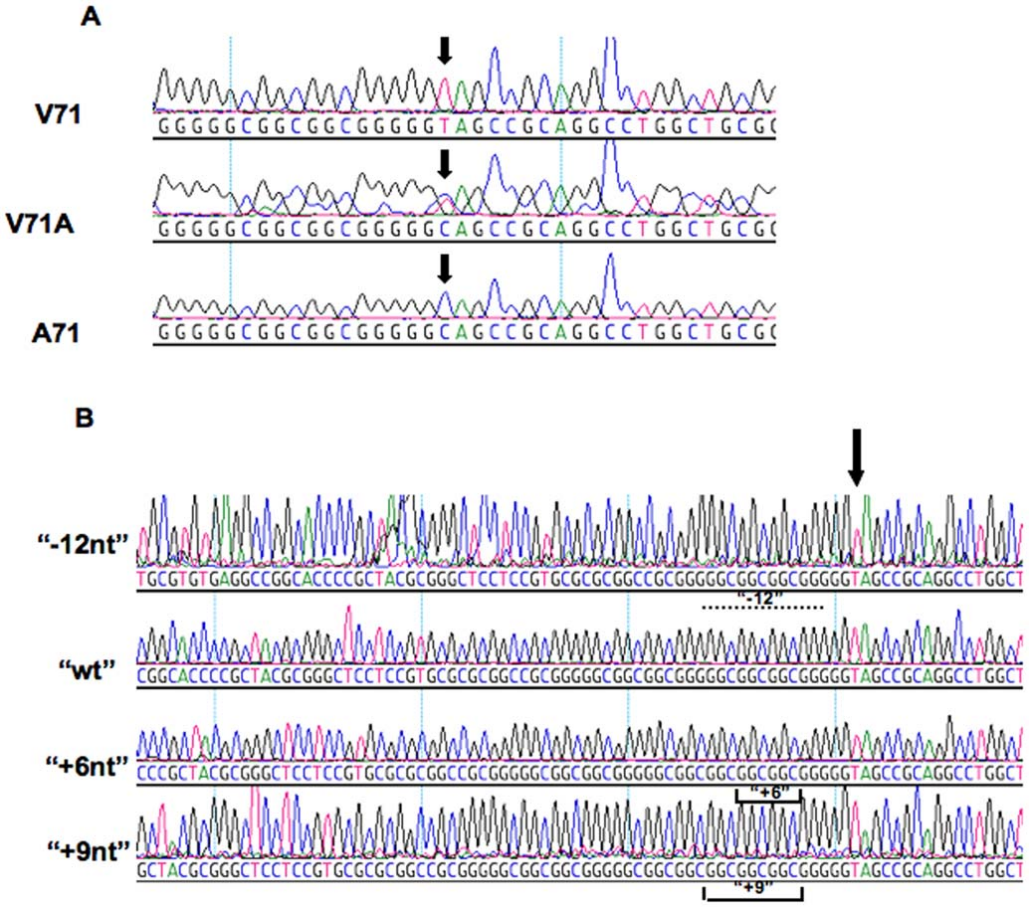
Sequences of Different Sheep *SPRN* alleles. (A) Electrophoregrams presenting the polymorphism V71A. Codon 71 is either GTA (Val) or GCA (Ala). (B) Electrophoregrams of sequencing reactions from cloned PCR products, representing a deletion of 12 bases, or insertions of either 6 or 9 bases relative to the wild type prototype.

With regards to the common SNPs, for codon 71 a total of 87 sheep (81.3%) were genotyped as T/T at nucleotide 212 (Val/Val), 3 sheep (2.8%) as C/C (Ala/Ala), and 17 sheep (15.9%) as C/T heterozygotes, with representative electropherograms of V71, A71 and V71/A71 heterozygotes presented in **Figure 3A**. The allele frequency for the T polymorphism is thus 89.3%. At nucleotide position 336 (silent polymorphism within codon 112), 52 sheep (48.6%) were genotyped as T/T, 19 sheep (17.8%) as C/C, and 36 sheep (33.6%) as C/T, giving an allele frequency of 65.4% for the T polymorphism. These data contribute to the assignment of V71(T) – Y112 (T) as the most common haplotype, *SPRN* ^a^, and V71(T) – Y112 (C) as the second most prevalent (*SPRN* ^b^: **Figure 2**).

#### Rare SNPs

With regard to less frequent variations, one Katahdin sheep was heterozygous A/G at nucleotide 61 defining an S21 sub-variant of *SPRN^c^* (i.e. one occurrence out of 214 alleles sampled; haplotype “*i*”, **Figure 2**). Three Cheviot sheep were heterozygous G/C at nucleotide 157. Inspection of co-inherited SNPs in codons 71 and 112, as well as cloning of individual PCR products defining a P53 sub-variant of *SPRN^b^* (i.e. three occurrences out of 214 alleles sampled; haplotype *j* and *k*, **Figure 2**). Three sheep (one Rambouillet, one Suffolk, one Katahdin) exhibited a missense C variation at nucleotide 377, H126P (haplotype *m*, **Figure 2**). The two former animals were homozygous C/C and the Katahdin was heterozygous (i.e. five occurrences out of 214 alleles sampled). All data from sequencing of bulk PCR products and cloning of individual PCR-amplified alleles are consistent with this SNP representing a sub-variant of *SPRN* ^a^. The last rare variant, a silent T polymorphism at nucleotide 396 (G132G) was represented by 3 heterozygous animals (one Cheviot, two Katahdin; i.e. 3 occurrences out of 214 alleles; haplotypes *n* and *o*, **Figure 2**), with all data from sequencing of bulk PCR products and cloning of individual PCR-amplified alleles being consistent with this rare SNP representing a sub-variant of *SPRN* ^b^. Thus the rare variant sighted most often (5/214 alleles) occurs in the framework of *SPRN* ^a^, the predominant haplotype and the prototype for the ancestral ovine *SPRN* sequence.

### Common Indel variations affecting the hydrophobic domain of ovine Sho

**Figure 4:**
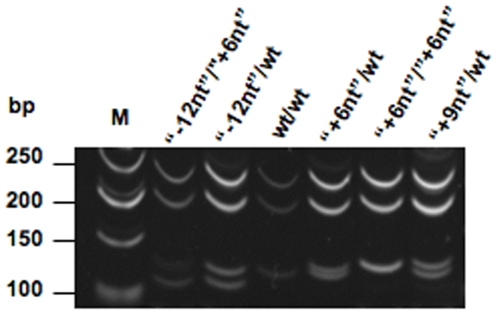
Detection of an Indel variation in the sheep *SPRN* open reading frame. Samples DNAs were amplified by PCR, digested with *Ava*I, and run on 15% PAGE gels. *Ava*I digestion generates 3 fragments: 251 bp, 204 bp, and variable sizes for the smaller fragments, corresponding to 118, 130, 136 and 139 bp respectively for “−12nt”, wild type, “+6nt” and “+9nt” allelic variants. M: Molecular weight marker.

Direct sequencing of amplified ovine genomic DNA revealed a number of samples with unusual electropherograms (not shown), wherein coherent sequence data diverged into a “mixed” output comprising two co-existing sequences. We inferred these data to be consistent with a common size variation in the length of the ovine *SPRN* ORF, with animals with mixed sequence electropherograms representing heterozygotes for size variation, and the boundary between coherent and mixed sequence in the electropherogram representing the boundary of the indel heterozygosity. This inference of genic size variation was confirmed in two ways. First, genomic DNA PCR products of the complete *SPRN* ORF were digested directly with *Ava*1 endonuclease to generate restriction fragments amenable to PAGE sizing. Representative analyses are shown in **Figure 4**. In contrast to invariant fragments of 252 and 204 nucleotides (nt) lying outside of the polymorphic region, size variation polymorphism was noted in smaller fragments corresponding to larger (“+6nt: and “+9nt”) and smaller (“−12nt”) variations from a wt restriction fragment length of 130 base-pairs. Secondly, we cloned and sequenced individual PCR products from 7 animals differing from the wt point of reference (established from allele frequency and alignment of multiple mammalian species), both to confirm the observation and to pinpoint the identity of the nucleotide variations. These data are shown in **Figure 3B** and can be explained in terms of expansion or deletion of nucleotides encoding a tract of hydrophobic amino acids in the centre of the protein. The prototype tract in the wt sequence commences after Arg58 and corresponds to three contiguous repeats (R1-R3) of the sequence Ala-Ala-Ala-Gly, a fourth polymorphic repeat R4 either Val-Ala-Ala-Gly or Ala-Ala-Ala-Gly, and a fifth degenerate repeat R5, Leu-Ala-Ala-Gly. Thus this central region of wt ovine Sho can be described in single letter code (starting at residue 54) by …GSSVR-[R1, R2, R3, R4, R5]-SSWR…

To assign the start and stop points in variants of this repeated sequence we compared the nucleotide sequences of repeats:
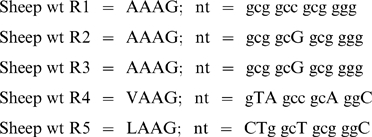
where the position of the valine in the V71A polymorphism is underlined. Whereas R1, R4, and R5 have unique nucleotide sequences, R2 and R3 are identical and thus cannot be distinguished from each other unless they occur together and are flanked by R1 and R4. The observed sequence variations in ovine *SPRN* alleles are all compatible with alterations within or adjacent to R3, represented as follows:
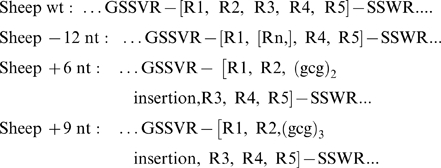



Thus in this scheme the “−12nt” variant corresponds to Δ67–70 (Genbank #EU380592). The “+6nt” sequence reported here and previously by Lampo *et al*, corresponds to insertion of two extra alanine codons to make a variant (Ala)_5_Gly repeat, and the “+9nt” sequence adds yet one more alanine codon to make a (Ala)_6_Gly variant of the (Ala)_3_Gly repeat prototype (Genbank #EU380590).

#### Frequency and breed distribution of ovine SPRN indel variants

**Figure 5:**
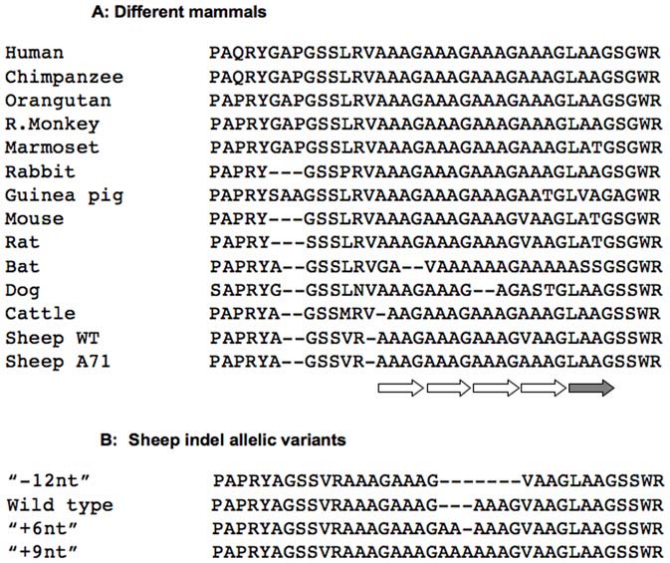
Alignment of Sho proteins. (A). Alignment of amino acid sequences (Clustal W) of the central regions of diverse Sho proteins. The hydrophobic domain area including amino acids 48 to 82 (according to the wild type sheep numbering) is presented. The four perfect (Ala)_3_Gly repeats are indicated underneath the sequences with open block arrows, while a fifth, degenerate repeat Leu(Ala)_2_Gly is indicated by a grey arrow. (B) Alignment of the sheep indel polymorphisms affecting the hydrophobic domain. For clarity, the wt sheep sequence is included in both types of alignment.

Using the above classification scheme in conjunction with genotyping by PAGE analysis of digested amplified DNA, and by cloning of individual PCR products we assessed the frequency of the indel variants in the sample of 214 ovine *SPRN* alleles. The sequence with four (Ala)_3_Gly repeats was most prevalent, with 166 occurrences (78%), in close agreement with the situation for wt alleles in other mammals (**Figure 5** **,** **Table 4**). On the other hand, one quarter of all sheep alleles sampled had expansions or contractions in this region: 43 occurrences (20%) of “+6nt” variants, 4 occurrences of “–12nt” variants, and a single occurrence of a “+9nt” variant **(** **Table 4** **)**. Breed distribution was assessed for the most common “+6nt” variant, but all breeds were found to include at least one heterozygote. For the rare indel alleles the sample size was insufficient to impart statistical power and here we merely note that the “+9nt” variant occurred in the single Navajo-Churro animal tested, and that 3 out of 4 of the “–12nt” polymorphisms occurred in Finn animals, with the fourth in the “composite” (Suffolk-Hampshire-Columbia) stock (**Table 2**).

#### Relationship of ovine SPRN indel variants to missense polymorphisms

Presence or absence of indel polymorphisms was used to construct coding region haplotypes (**Figure 2** **)**. The common “+6nt” polymorphism was present in conjunction with the common missense variation noted above (i.e. T-T, and T-C) where T and C refer to alternative nucleotides in codons 71 and 112 defining the ovine *SPRN* types *a* and *b* (**Figure 1** **,** **2**). Thus the “+6nt” polymorphism may have arisen independently in the most common *SPRN a* and *b* haplotypes to give the corresponding “+6nt versions” (i.e., alleles *c* and *d*, respectively), or may have occurred once in an *a* or a *b* haplotype, and then been redistributed by a crossover event (discussed in greater detail below). All 4 “–12” polymorphisms identified occurs in framework like the *b* haplotype (e). Haplotype *h* was assigned for the single “+9” allele identified to date. Our haplotypes *a*–*g* constructed by excluding rarer SNPs (as described above) exhibit limited overlaps with haplotypes based upon analysis of Cheviot, Welsh Mountain and undefined “modern” breeds [17]. Specifically, while haplotype *a* corresponds to haplotype “2”, *b* to “1”, *d* to “7”, and *f* to “11”, haplotypes *c*, *e* and *g* are not represented in the dataset of Stewart *et al*. Also, and as implied by the nomenclature system applied here, we found the most common haplotype to be haplotype *a*. and this to correspond to a T residue at nucleotide 336 in the silent polymorphism Y112Y (56% of animals).

### Allelic forms of ovine Sho proteins assessed in Neuroblastoma cells

**Figure 6:**
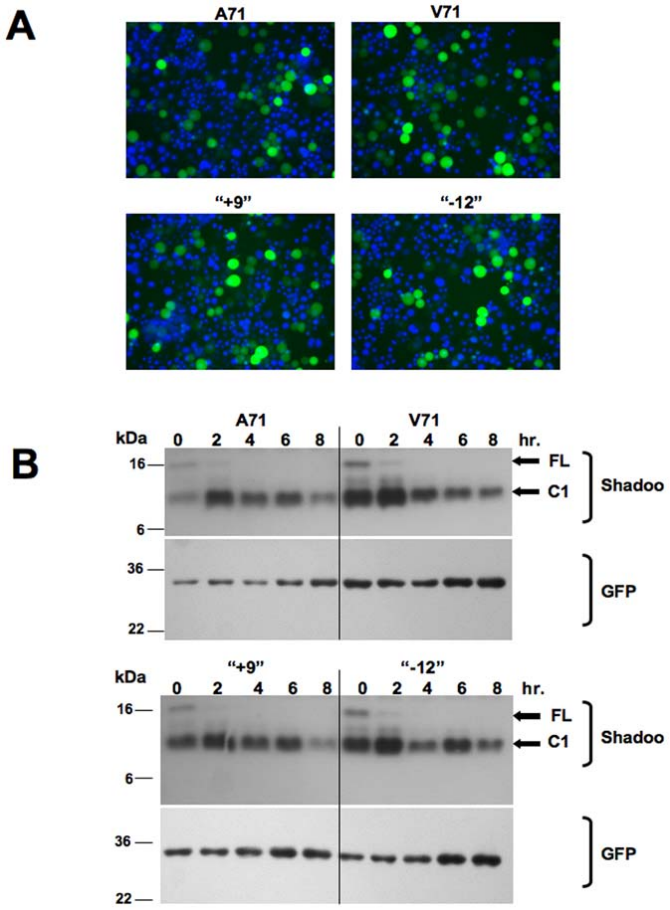
Expression and turnover of Sheep Sho alleles in transfected N2a cells. (A) GFP fluorescent signals in fields of transfected cells to demonstrate similar transfection efficiencies. Individual values were V71 (10.1%), A71 (5.1%), “+9” (8.7%), “−12” (7.1%). (B) Western blot analysis of N2a transfected cells chased for different time periods in the presence of cycloheximide. Proteins samples are de-glycosylated with PNGase F. Positions of full-length and C1 fragments of sheep Sho are indicated, detected with a C-terminal anti-Sho antibody.

To assess if ovine indel *SPRN* alleles produce stable proteins or proteins with distinct biochemical properties we performed acute transfections into N2a neuroblastoma cells using a bigenic “pBUD” expression vectors encoding Sho alleles of interest plus a GFP reporter gene driven from a separate promoter. Cycloheximide was added to some samples to prevent *de novo* protein synthesis. Proteins in cell lysates were assessed by immunoblot with a monospecific ‘06Sh3a’ antiserum [11] raised against a C-terminal peptide epitope in mouse Sho (**Figure 6**).

To appraise potential confounding effects of variable transfection efficiency, absolute transfection efficiencies were established for each plasmid by robotic cell counting, by comparing the number of nuclei stained with Hoechst dye versus the total number of GFP-positive cells. These experiments failed to reveal differences between the four alleles or the empty vector control expressing just GFP (average 3%, range 2.3 −3.5%: **Fig 6a**). In accord with these data, robust and similar immunoblot signals for Sho protein were seen in cells transfected with the 4 alleles (**Fig. 6b**) (prior experiments have demonstrated that Sho immunoreactivity is not detected in untransfected cells [11]). Probing with an antibody against GFP comprised a control for plasmid-based expression and sample loading. Time-point data establish that sheep Sho proteins are similarly labile, with a half-life measured at under 2 hours (compare 0 and 2 hr. measurements). They also establish that sheep Sho protein expressed in N2a cells is subjected to a “C1-like” processing event, irrespective of allelic type. This effect is similar to that seen for mouse Sho, and could prove analogous to the endoproteolytic processing of PrP.

## Discussion

### Human and Mouse versus Ovine SPRN

In this study, we assessed polymorphisms of the Sho gene of humans, mice and sheep. We find that mice have little coding sequence variation in this gene, in accord with a recent study based on a different sample set [18]. In the case of human *SPRN* we confirmed a signal peptide M7T variation was found in our sample of Caucasian DNAs (and as reported previously by others [19]), but we were unable to define a high frequency polymorphism within the boundaries of the mature human Sho protein (residues 24 to 126). While this picture might change with the inclusion of larger sample sizes and samples from different ethnic groups, the compiled NCBI SNP database is also not supportive of a plethora of missense variation within human *SPRN*, even though this includes samples derived from multiple ethnic groups (HapMap project, including populations that have Asian, African and European origins). Other described human *SPRN* SNPs comprise a signal peptide polymorphism L13Q and the coding sequence variation G42 (SNPs rs2492667 and rs2480253, respectively), though neither was found in our sample of predominantly Caucasian DNAs. Our data do, however, offer a parallel to the pattern of variation in human *PRNP*. As assessed by genotyping the HGPD-CEPH repository, *PRNP* variation is dominated by a single common M129V polymorphism with an overall V129 allele frequency of 23% (rising to 65% V129 in American populations). The other polymorphisms are all rare, including E219K polymorphism (K219 1.3% overall, rising to 5.4% in Pacific populations), insertions and deletions in the octarepeat region (0.4%), plus a miscellany of silent substitutions (0.03%) and missense SNPs (142Ser and 232Arg totalling 0.03% alleles) [20]. Indeed, overall nucleotide diversity in human *PRNP* has been estimated at three times lower than an average derived from scrutiny of 245 other genes [20]. Standing in apparent contrast to these observations, ovine *SPRN*, like ovine *PRNP*, exhibited a number of coding sequence alterations. Insofar as three of the more common haplotypes *a–g* were absent from another study [17] our data may yet underestimate the full extent of diversity in *SPRN*, lending further support to the main conclusion of this paper. Parenthetically the trivial technical explanation that ovine *SPRN* variants defined here and by others derive from a pseudogene can be excluded because (i) current versions of the sheep genome do not provide evidence for such a pseudogene, and (ii) sequence variations described herein are all in-frame and lie within the context of a complete, contiguous ORF.

### An unstable DNA tract in the ovine SPRN gene?

Variations in ovine *SPRN* are notable from the perspectives of the DNA sequence, and of the predicted chemical properties for the variant proteins. With regards to the former, while SNP variations require little comment, we defined three indel variations in addition to the wild type norm, all occurring in a 41-nucleotide tract that is comprised solely of G/C base-pairs. Because our analyses used replicate PCR reactions of starting genomic DNA, high fidelity polymerases, plus sequencing and restriction mapping of total PCR products, we can exclude that the polymorphisms reflect rare *in vitro* polymerase errors captured by molecular cloning. More likely, the polymorphisms reflect *bona fide* germ-line replication errors prompted by stable hairpin structures arising as a consequence of the extreme G+C content. The “+6nt” and “+9nt” alleles described here are trinucleotide expansion within alanine codons, while “-12nt” represents loss of a complete (Ala)_3_Gly unit (**Figure 5B**). In the case of trinucleotide repeat diseases, normal variation in a mean repeat size stands in contrast to unbridled instability (expansion) once a threshold size for a pre-mutation has been exceeded. For CAG trinucleotide repeat diseases the range of normal variation is from 4–19 to 6–44 repeat units rising to 10–33 and 39–82 repeats respectively for pathogenic alleles [21], but in the case of pure polyalanine tracts (below) the percent increase from a normal to pathogenic number ofrepeats may be smaller. At this stage we suggest that scrutiny of more sheep samples will reveal yet further size polymorphisms.

### G+C content of SPRN hydrophobic tract residues: species differences

The ovine DNA coding sequence for the Sho hydrophobic domain encompasses a 41 nucleotide tract (42 nucleotides in the case of alleles encoding 71Ala) composed entirely of G+C base-pairs, yet in the nuclear genetic code alanine and glycine are each encoded by four codons, and can therefore include A or T residues in the third position. Indeed, alignment of sequences encoding the Sho hydrophobic domain from different mammals reveals usage of codons with A or T at the third position, with 1/41 (cattle), 3/41 (chimpanzee), 4/41 (humans), 6/41 (rhesus monkey) or 9/41 (mouse, rat) A or T nucleotides interposed into this tract. If this analysis is corrected to consider a shorter contiguous tract (starting at Ala 58 of the sheep sequence, as sheep and cows differ from most other mammals by a codon deletion, having a first repeat of Val(Ala)_2_Gly instead of Val(Ala)_3_Gly: **Figure 5A**) then these figures become 0/32 (cattle), 1/32 (chimpanzee), 2/32 (humans), 4/32 (rhesus monkey) or 8/32 (mouse, rat) A or T nucleotides interposed within a 32 nucleotide tract. Within this dataset of mammalian *SPRN* sequences, tracts of uninterrupted G+C base-pairs are a special feature of the ovine and bovine gene, perhaps contributing to species differences in the occurrence of hydrophobic domain coding sequence expansion and contraction. For trinucleotide-repeat motifs, the ability of individual DNA strands to form hairpin structures during replication and repair comprises a common postulate within all hypotheses to account for variations in unit length. High GC content determining melting temperature, as well as the inclusion of “hairpin-prone” CGG units in alanine tracts may thus predispose the central coding region of ovine *SPRN* to length variation [22].

### Properties of Sho proteins with different hydrophobic domains

Hydrophobic region polymorphisms of ovine Sho protein are summarized in **Figure 5B**. While some of these polymorphisms involve conservative amino acid replacements (e.g. Ala71Val) and are documented in healthy (albeit young) animals, their phenotypic impact in aged or infected animals remains to be determined. The possibilities are that they could modulate prion infections, or in concert with other factors (e.g. greater degrees of genomic instability), might contribute to spontaneous prion disease in aged animals. In human *PRNP*, the widely-studied A117V mutation, and a variety of synthetic alanine to valine mutations at 4 other positions in the hydrophobic tract increase biogenesis of a transmembrane form of the protein (“CtmPrP”), and may be a cause of Gerstmann–Straussler Syndrome and neurodegenerative disease in transgenic mice, respectively [23]. In this vein, the human *SPRN* polymorphism T7M is now reported to be associated with sporadic CJD with a p value of 0.009 [19]. To the best of our knowledge the only mechanistic effect of PrP polymorphisms in the N-terminal signal peptide (described by Stewart *et al* for PrP L9R) is to alter the formation of the topological form called Ctm PrP [24], and interestingly this form involves embedding of the central hydrophobic tract into the plasma membrane. Perhaps a similar effect may explain how a human *SPRN* signal peptide polymorphism can alter some aspect of the pathogenesis of sporadic CJD.

Also worthy of consideration, there are connections between expansions in homopolymeric alanine and several neurodegenerative diseases. The first theme comprises trinucleotide expansions of genomic DNA. Here, typically occurring in alanine tracts of transcription factors, expansions above a normal range containing from as little as 1 residue to 10 extra alanine codons underlie at least 9 human conditions [25], [26]. Interestingly, the proteins with alanine expansions have a “gained” tendency to aggregate versus their wt counterparts [27], [28]. The second theme of note is a tendency for alanine-rich sequences to undergo α-helical to β structure transitions [29], [30], suggesting that structural studies of ovine Sho protein with wt and variant (Ala)_3_Gly repeat arrays may prove fruitful.

### Parallel variation in Ovine SPRN and PRNP?

Diverse coding sequence variations in both ovine *SPRN* and *PRNP* raises the broader questions of origin, and the types of selection pressures that might operate on these genes. In terms of DNA replication mechanisms that might predispose to sequence variation, there is unlikely to be a single common theme: *PRNP* is characterised by missense mutations whereas *SPRN* is characterised by indel variation in a G/C rich tract as well as missense variation. With regards selection pressure, heterozygosity for the *PRNP* M129/V129 missense polymorphism (a genotype disfavouring prion replication) was attributed to a selective advantage against cannibalistic acquisition of prion infectivity in prehistoric humans [31]. However, others have raised the issue of ascertainment bias affecting this conclusion [32], [33]. For exposure to prion disease to shape *PRNP* allele frequency in susceptible populations, salient parameters will be the presence of a disease-related physiological deficit at the age when mating partners are selected, and exposure at an endemic level. Perhaps more plausibly, pressures to retain certain *PRNP* haplotypes (actually the common ARQ haplotype that is found to confer heightened susceptibility to common scrapie isolates, versus resistance-associated ARR or AHQ haplotypes of *PRNP*) may reflect selective advantages acting at the time of birth or during early postnatal life [8]. PrP^C^ expression is increased during instances of brain trauma [34] and PrP^C^ is associated with neuroprotective activity against a number of toxic compounds. Interestingly, in differentiated neuronal cultures, PrP^C^ and Sho exert similar protective effects against a toxic stimulus [11]. Altered interactions with a common partner such as L_PrP_ [35] deriving from selection for an altered form of PrP might in turn select for a compensatory allelic form of Sho. Another possibility is that common genetic variation in ovine *SPRN* driven by an unstable DNA tract will apply selection pressure for PrP variants with altered physiological activity to offset the action of the *SPRN* variants. However, as the dataset for genome wide variation in sheep (i.e. behaviour at other “control” loci) in sheep lags behind the situation in humans, and as the hypothetical L_PrP_ protein waits to be identified, these possibilities must be considered as speculative for the time being.

### Summary

Our studies define abundant coding sequence variation in the ovine *SPRN* gene. Allelic variants are analyzed by breed, and some approach a frequency of 20%. Most variation occurs in a hydrophobic tract encoded by 5 tandem repeats, and four alleles varying in this region engender stable proteins that may in turn determine different biological responses. Further studies on the transmission genetics of the *SPRN* indel alleles and the *SPRN+PRNP* genotypes of scrapie-challenged animals may be enlightening.

## Supporting Information

Table S1 (0.06 MB PDF)
