# Supplementary material for: Frequent Missense and Insertion/Deletion Polymorphisms in the Ovine Shadoo Gene Parallel Species-Specific Variation in PrP
Source: PLoS One. 2009 Aug 6;4(8):e6538. doi: 10.1371/journal.pone.0006538 (PMC2716538; doi:10.1371/journal.pone.0006538)
Supplement: Table S1 — (0.06 MB PDF) [file pone.0006538.s001.pdf]

Table S1

Table S1: primers

name	sequence
SF1	5’-TTCCAGACCCTCACCCGCTTCCTT-3’
SR1	5’-CTGCTCACCACACTGGGTCTCTGTT-3’
SF2	5'-TTCCCAGCTCCTGAGCGCCC-3'
SR2	5'-TGGGGAGAGGGCACAGGCTG -3'
HF2	5’-GCCTTGAGCCCTCCGCCC-3’
HR2	5’-GCGTGG GCAGGACGGTGG-3’
HF1	5’-CTCTCCAGCCTTGAGCCCTCCGCCC-3’
HR1	5’-TGATGTCGCCTCCCTGCCCGTGAGTC-3’
MF1	5'-TGCACAGAAGCTGAA GCCC-3'
MR1	5'-ACAGGCTAGGAGCTAGACC-3'
SF3	5’-GCAAGCTTGGTACCAGGAACATGAACTGGGC-3’
SR3	5'-TATCTAGATCACTAGGGCCGCAGCAGCCGCAG-3'
